# Supplementary material for: Absence of MBP 3’ UTR in Mice Disrupts mRNA Transport, Myelination, and Motor Learning
Source: bioRxiv. 2025 Dec 25:2025.12.23.696264. Preprint. [Version 1] doi: 10.64898/2025.12.23.696264 (PMC12776054; doi:10.64898/2025.12.23.696264)
Supplement: Supplement 1 [file media-1.zip › Mbp mRNA Manuscript_SUPPLEMENTAL FIGURES.docx]

**SUPPLEMENTAL FIGURES**

**Figure S1. *Mbp* mRNA colocalizes with tubulin and actin**

(A) Confocal micrographs (60X) of DIV 1 and 2 immunopanned rat primary oligodendrocytes immunostained against tubulin, stained for actin using phalloidin, and smFISH against *Mbp* mRNA.
(B) *Mbp* mRNA pixel area measured in concentric rings around the cell nucleus

(Two-way ANOVA with Šídák's multiple comparisons test, n=3 individual experiments, 14–18 cells per DIV).

**Figure S2. P14 and P30 qPCR for *Mbp* mRNA in different brain regions**

(A) Relative *Mbp* mRNA expression from P14 mice in the whole brain (P=0.0302, Student’s t-test, n=3 mice per genotype).
(B) Relative expression of *Mbp* CDS from P30 mice in corpus callosum (P=0.06, 0.012, One-way ANOVA with Tukey’s multiple comparisons test, n=2-3 mice per genotype).
(C) Relative expression of *Mbp* CDS from P30 mice in the spinal cord (P=0.04, <0.0001, One-way ANOVA with Tukey’s multiple comparisons test, n=3 mice per genotype).
(D) Relative expression of *Mbp* 3' UTR from P30 mice in the corpus callosum (P=0.04, 0.002, One-way ANOVA with Tukey’s multiple comparisons test, n=2-3 mice per genotype).
(E) Relative expression of *Mbp* 3' UTR from P30 mice in the spinal cord (P=<0.0001, <0.0001, One-way ANOVA with Tukey’s multiple comparisons test, n=3 mice per genotype).
(F) *Mbp* CDS primer efficiency (R^2^=0.983).
(G) *Mbp* 3’ UTR primer efficiency (R^2^=0.960).
(H) *Gapdh* primer efficiency (R^2^=0.981).
(I) *Hgprt* primer efficiency (R^2^=0.997).
(J) Relative expression of *Mbp* CDS in the whole brain at P14 (P=0.0302, Student’s t-test).

Data represents mean ± SEM. *P < 0.05, **P < 0.01, ***P < 0.001, ****P< 0.0001.

**Figure S3. *Mbp* mRNA colocalizes with lysosomes in homozygous *Mbp* 3’ UTR KO mouse oligodendrocytes**

(A) 60X confocal micrographs of immunopanned DIV4 mouse primary oligodendrocytes cultured from *Mbp* 3’ UTR KO mice. smFISH was performed to visualize *Mbp* mRNA and immunostained for lysosomal marker LAMP1.

**Figure S4. Myelin staining of P21 and P90 mouse brains**

(A) MBP immunohistochemical staining of a P21 mouse brain imaged at 10x magnification. Dashed white line indicates the brain boundary.
(B) High-magnification (60X) image from P21 cerebellum stained for MBP.
(C) Max projection of confocal micrograph (60x) from cerebellum of P90 mouse brain immunostained for MBP and CNP proteins (n=3 mice per genotype).
(D) CNP intensity quantification in hi-mag cerebellum image from P90 mouse brain (P=0.29, Student’s t-test).
(E) CNP area quantification in hi-mag cerebellum image from P90 mouse brain (P=0.53, Student’s t-test).

Data represents mean ± SEM. *P < 0.05, **P < 0.01, ***P < 0.001, ****P< 0.0001.

**Figure S5. *Mbp* 3’ UTR KO have intact myelination of the sciatic nerve**

(A) 3-month-old mouse sciatic nerve EM (2000x).
(B) Scatterplot of axon caliber vs. g ratio. Each data point represents one myelinated axon (n=3 mice per genotype, 30–97 axons per image).
(C) Sciatic nerve g ratio (P=0.96 Student’s t-test).
(D) Myelinated axon caliber (P=0.35 Student’s t-test).
(E) Percent of unmyelinated axons. (P=0.37 Student’s t-test).

Data represents mean ± SEM. *P < 0.05, **P < 0.01, ***P < 0.001, ****P< 0.0001.

**Figure S6. *Mbp* 3’ UTR KO mice do not exhibit differences in the open field assay**

(A) Schematic of open field assay. Mice were allowed to move freely through an arena for 2 hours. Red dashed line represents the “center,” defined as the middle 50% of the arena.
(B) Representative activity traces for open field assay of wildtype (n=12 mice) and *Mbp* 3’ UTR KO mice (n=10 mice).
(C) Distance traveled (P=0.76, 0.42. One-way ANOVA with post hoc Dunnett’s multiple comparisons test).
(D) Percent of distance traveled binned by 15 minutes (P=0.80 time x genotype interaction. Two-way ANOVA with post hoc Dunnett’s multiple comparisons test).
(E) Center entries (P=0.38, 0.57. One-way ANOVA with post hoc Dunnett’s multiple comparisons test).
(F) Percent of center entries binned by 15 minutes. (P=0.98, time x genotype interaction. Two-way ANOVA with post hoc Dunnett’s multiple comparisons test).
(G) Percent of time in center (P=0.65, 0.86. One-way ANOVA with post hoc Dunnett’s multiple comparisons test).
(H) Percent of time in center binned by 15 minutes. (P=0.28, time x genotype interaction. Two-way ANOVA with post hoc Dunnett’s multiple comparisons test).

Data represents mean ± SEM. *P < 0.05, **P < 0.01, ***P < 0.001, ****P< 0.0001.
